# An immobilized biosorbent from *Paenibacillus dendritiformis* dead cells and polyethersulfone for the sustainable bioremediation of lead from wastewater

**DOI:** 10.1038/s41598-023-27796-w

**Published:** 2023-01-17

**Authors:** Ghada E. Dawwam, Nehad M. Abdelfattah, Mohamed O. Abdel-Monem, Hossam S. Jahin, Amal M. Omer, Khadiga A. Abou-Taleb, Eman S. Mansor

**Affiliations:** 1 Botany and Microbiology Department, Faculty of Science, Benha University, Benha, 13518 Egypt; 2 Central Laboratory for Environmental Quality Monitoring, National Water Research Center, Elkanatir, 13621 Egypt; 3 Department of Soil Fertility and Microbiology, Desert Research Center, El-Matareya 11753, Cairo, Egypt; 4 Department of Agricultural Microbiology, Faculty of Agriculture, Ain Shams University, Hadayek Shubra 11241, P.O. Box 68, Cairo, Egypt; 5 Water Pollution Research Department, National Research Centre, Environment and Climate Change Research Institute, Dokki, Cairo, Egypt

**Keywords:** Microbiology, Chemistry

## Abstract

Heavy metals, including lead, cause serious damage to human health and the surrounding environment. Natural biosorbents arise as environmentally friendly alternatives. In this study, two of the 41 isolates (8EF and 17OS) were the most efficient bacteria for growing on media supplemented with Pb^2+^ (1000 mg/L). At high concentrations up to 2000 mg/L, the pioneer isolate 17OS exhibited remarkable resistance to multiheavy metals. This isolate was identified as *Paenibacillus dendritiformis* 17OS and deposited in GenBank under accession number ON705726.1. Design-Expert was used to optimize Pb^2+^ metal removal by the tested bacteria. Results indicated that four of six variables were selected using a minimum-run resolution IV experimental design, with a significant affecting Pb^2+^ removal. Temperature and Pb^2+^ concentration were significant positive influences, whereas incubation period and agitation speed were significant negative ones. The tested strain modulated the four significant variables for maximum Pb^2+^ removal using Box–Behnken design. The sequential optimization method was beneficial in increasing biosorption by 4.29%. Dead biomass of *P. dendritiformis* 17OS was embedded with polyethersulfone to get a hydrophilic adsorptive membrane that can separate Pb^2+^ easily from aqueous solutions. SEM images and FT-IR analysis proved that the new biosorbent possesses a great structure and a lot of surface functional groups with a negative surface charge of − 9.1 mV. The removal rate of 200 mg/L Pb^2+^ from water reached 98% using 1.5 g/L of the immobilized biosorbent. The adsorption isotherm studies were displayed to determine the nature of the reaction. The adsorption process was related to Freundlich isotherm which describes the multilayer and heterogeneous adsorption of molecules to the adsorbent surface. In conclusion, dead bacterial cells were immobilized on a polyether sulfone giving it the characteristics of a novel adsorptive membrane for the bioremediation of lead from wastewater. Thus this study proposed a new generation of adsorptive membranes based on polyethersulfone and dead bacterial cells.

## Introduction

Toxic heavy metal ions that resulted from intensive industrialization, modern farming techniques, military actions, and weathering processes have become one of the most significant contaminants in water supplies^1^. Contrary to other toxic materials, heavy metal ions are accumulated in tissues of living organisms and aren't biodegraded in nature. Despite their widespread use, heavy metals can cause substantial toxicity in living creatures^2^. Lead, copper, mercury, arsenic, and chromium can all have detrimental effects on the skin, kidneys, liver, and lungs^3^. Lead (Pb^2+^) is very dangerous due to its toxicity and environmental dissemination^4^. It can stop the action of enzymes and proteins, replace critical cells’ ions (Mg^2+^, Ca^2+^, Na^+^, and Fe^2+^), and impede Ca^2+^ transport, so Pb^2+^ is regarded as carcinogenic and very poisonous. Furthermore, Pb^2+^ accelerates reactive oxygen species generation, which results in oxidative stress, and damages cell^5^. Inhalation of Pb-polluted dust, as well as ingestion of contaminated food or water, causes Pb^2+^ to be absorbed and disseminated into human tissues^6^.

Membrane technology, precipitation, activated carbon adsorption, and ion exchange are common physicochemical methods for removing toxic metals from wastewater^7^. Many of the common drawbacks of conventional processes to remove heavy metals, such as excessive toxic sludge production and low-quality treated water, can be overcome if membrane technology is combined with adsorption technology (Adsorptive Membrane Technology). This membrane offers a number of characteristics such as lack of phase change or chemical additives, flexibility and ease of scaling up, simplicity of idea and operation, energy efficiency, and small process footprint^8^. Adsorption is the most popular heavy metal removal technique due to its simplicity, versatility, low cost, and eco-friendliness. However, adsorption has some disadvantages, including a sluggish rate and a high internal diffusion resistance^9^. Additionally, the efficiency provided by the adsorption method often tends to decline after repeated use^10^.

Microorganisms that have been immobilized onto the proper substrates can be used in a variety of procedures and are simple to recover and reuse. Immobilization can increase the operational stability of cells, shield them from the impacts of high pH, poisonous substances, and violent reaction technologies, and lower the possibility of contamination of cell cultures^11^. Mixed matrix membranes (MMMs) are frequently made using polymers as polyethersulfone (PES), polysulfone (PSf), polyvinylidene fluoride (PVDF), and polyacrylonitrile (PAN)^12^. Out of these, PES is used on a large scale in the phase inversion fabrication of polymer membranes for microfiltration, gas separation, and ultrafiltration due to its great mechanical strength and chemical stability, and low cost as a commercial polymer^13^.

Bacteria are more effective in adsorbing harmful metals, especially at low concentrations in solutions^14^. Huma et al.^15^ indicated that *Bougenvillae spectobilisis* can be effectively used to remove copper and cadmium ions from an aqueous medium. In addition, citric acid was used to modify the biosorbent *B. spectabilis* for the biosorption of Pb^2+^ ions from an aqueous medium. Citric acid-modified *B. spectabilis* showed a noticeably higher capacity for biosorbing Pb^2+^ ions from an aqueous medium than unmodified *B. spectabilis*^16^. One distinguishing feature of biosorbents is that they can be alive or dead. To simplify complexity, most investigations on metal elimination employ dead biosorbents as the favored alternative^17^. Autoclaving a bacterial biosorbent improves its capacity for heavy metal biosorption^18^, possibly because the cell wall is degraded, having possible binding sites that can accommodate more metal ions. Also, live biosorbents possess a unique collection of benefits. They can transport adsorbed heavy metals inside cells and change the nature of heavy metal ions to minimize toxic effects^19^. However, only a few studies have evaluated the capability of dead and life biosorbents to adsorb hazardous heavy metals.

This study aimed to (1) isolate Pb-tolerant bacteria from different contaminated heavy metal sites, (2) identify and characterize the most potent isolate, (3) determine the minimum inhibitory concentration (MIC) and maximum tolerance concentration (MTC) for bacteria, (4) Optimize heavy metal removal by the tested isolate using Design-Expert, and (5) a new biosorbent technique needed to be achieved by isolation of lead tolerant bacteria and immobilizing them on PES.

## Materials and methods

**Figure 1:**
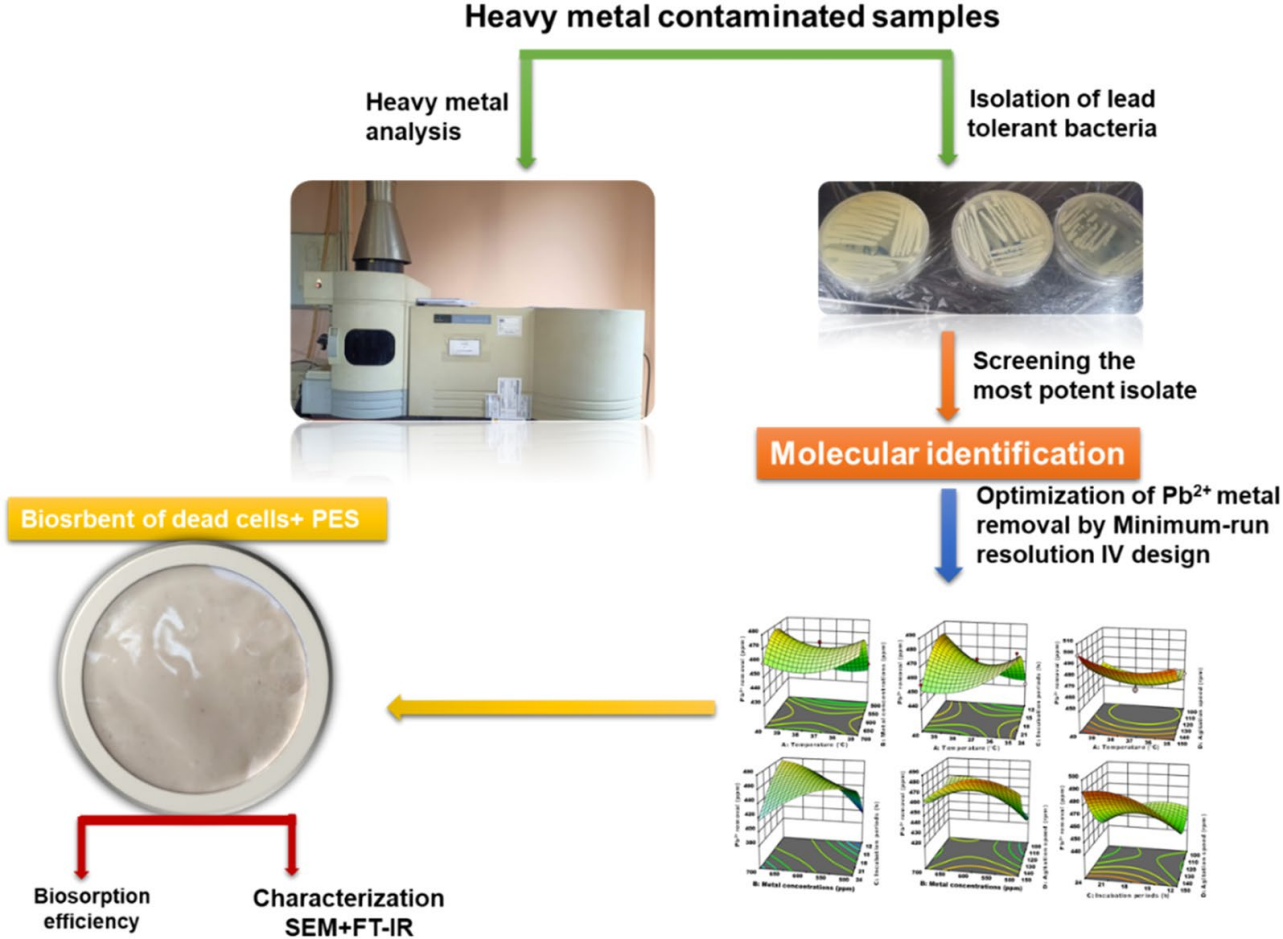
Graphical abstract for lead- tolerant bacteria under investigation.

An overview of the work done in this study is depicted in Fig. 1. Lead-tolerant bacteria were isolated from different contaminated sites. These bacteria were screened for the most potent isolate that was identified using 16S rRNA. Screening of the most significant variables affecting Pb^2+^ removal by bacterial isolate was studied by minimum-run resolution IV design. The most Pb^2+^ tolerant bacterium was encased in PES. Biosorption efficacy and different physicochemical characteristics of the biosorbent membrane were determined.

### Sample collection

Four samples were collected from different sites contaminated with heavy metals [electrical factory (EF), oil and soap company (OS), gas station (GS), and soil near sewage water (SW)] at Qalyubia Governorate (30°18′0″N/31°15′0″E) in Egypt. These samples served as a source for isolating heavy metal adsorbent bacteria. These samples were kept at 4 °C until analysis.

### Heavy metal analysis

Analysis of different heavy metals presented in four samples was carried out using inductively coupled plasma-optical emission spectrometry (ICP-OES; Optima 5300 DV; Perkin-Elmer, USA), as shown in Table 1. Analyses were carried out in an ISO 17,025:2017 certified laboratory of the Central Laboratory for Environmental Quality Monitoring, National Water Research Center (Egypt).

**Table 1:** Analysis of heavy metals with ICP (mg/L) in collected samples.

Samples		Heavy metal concentrations (mg/L)												
		Al^+3^	Ba^+2^	Cd^+2^	Cr^+3^	Co^+2^	Cu^+2^	Fe^+3^	Pb^+2^	Mn^+2^	Mo^+2^	Ni^+2^	Sr^+2^	Zn^+2^
Water	EF	–	0	–	–	–	0.14	0.02	0.01	1.9	0.02	9.36	0.05	0.19
Soil	OS	38.6	0.7	0	0.07	0	0.09	32.9	0.05	0.77	0.02	0.07	0.42	0.21
	GS	14.1	0.4	0	0.09	0	0.15	52.4	0.09	0.76	0.1	0.05	0.76	0.37
	SW	118	1.2	–	0.34	0.2	0.49	192	0.12	4.66	0.02	0.32	0.59	0.86

Sb^+3^, As^+3^, Se^−2^, Sn^+2^, V^+2^, and Hg^+2^ were not detected. *EF* Electrical factory, *OS* Oil and soap company, *GS* Gas station, and *SW* Soil near sewage water. – , Not detected.

Wastewater and soil analyses for different heavy metals were carried out according to^20^ and^21^ respectively. The wastewater sample was digested in nitric acid (HNO_3_), whereas soil samples were digested using concentrated HNO_3_, HCl, and HF. The digested solutions were filtered by filter papers with a pore size of 2.5 µm, and the volume was supplemented to 50 ml with deionized water. These solutions were analyzed for heavy metals using ICP-OES.

To minimize error, metal analysis was performed while using blanks. Triplicate measurements and analyses of verified reference materials for every metal (Merck) were typically included for quality assurance, as per ISO/IEC 17025 for laboratory accreditation. At five different concentrations (1000, 500, 100, 50, and 25 µg/L), a test of heavy metal recovery was conducted. The typical relative standard deviation was < 5%.

### Isolation and screening for Pb^2+^-tolerant bacterial isolates and maintenance of cultures

To start, 10 g or 10 ml of soils or effluent samples were enriched in Erlenmeyer flasks (150 mL in volume) containing 90 ml nutrient glucose broth medium (involving 10.0 g/L glucose, 5.0 g/L peptone, and 3.0 g/L beef extract and adjusted pH to 7.0) for 72 h at 30 ± 2 °C using shake flasks at 150 rpm. After incubation, the turbid medium was diluted by sterilized distilled water to 10^−5^ and subcultured on a solidified nutrient glucose medium supplemented with 1000 mg/L Pb (NO_3_)_2_. Different colonies were picked, purified on the previously mentioned medium, and stored at 4 °C for further study^22^.

### Evaluation of MIC and MTC for the most tolerant isolates

Using the approach outlined by Khan et al.^23^, the glucose broth medium was supplied with different Pb^2+^ concentrations (500, 1000, 1600, 1800, 2000, 2250, 2500, 2750, 3000, and 3250 mg/L). Selected isolates were inoculated into the previously mentioned medium and incubated for 72 h at 30 ± 2 °C. After the incubation period, 10 mL broth samples were taken from bacterial growth cultures to calculate the optical density (O.D) of growth using an SV 1100 digital visible spectrophotometer at 620 nm. MTC is the greatest concentration at which bacterial isolates can grow, and MIC is the lowest concentration that entirely prevents bacterial growth.

### Multimetal resistant detection

The bacterium with the highest MIC value for Pb(II) was chosen, and its resistance to other hazardous heavy metal ions was assessed using a multimetal resistance test. Using the glucose broth dilution method, resistance research against Cr(VI), Cd(II), Zn(II), and Cu(II) was conducted. The growth of metal tolerance bacteria was assayed as described above.

### Bacterial identification and phylogenetic analysis

The most active bacterial isolate (17OS) was identified based on morphological appearance under the microscope and the cultural and biochemical properties according to the keys proposed by Vos et al.^24^. The 16S rRNA of the 17OS isolate was amplified using polymerase chain reaction (PCR) using the following primers: 16S-F (forward, 5′-AGAGTTTGATCMTGGCTCAG-3′) and 16S-R (reverse, 5′-TACGGYTACCTTGTTACGACTT-3′^25^. This was performed to verify the identity of the bacterial strain. Each 20 μL PCR mixture contains 10 μL of 2 Es Taq Master Mix, 1 μL forward primer, and 1 μL reverse primer. The steps included in the PCR programming were as follows: primary denaturation for 15 min at 94 °C, secondary denaturation for 30 s at 94 °C, annealing for 1 min at 56 °C, extension for 1 min at 72 °C, and final extension for 5 min at 72 °C. The PCR products were purified using a DNA purification kit from Qiagen, Inc. (Valencia, CA, USA) and examined using horizontal electrophoresis on a 1% agarose gel.

Using an automated DNA sequencer (Applied Biosystems 3130 genetic analyzer; Applied Biosystems Foster City, CA, USA), the forward and/or reverse directions of a purified PCR product were sequenced. To determine the sequence identity to GenBank accession, a Basic Local Alignment Search Tool (BLAST^®^) analysis was carried out^26^. The resulting sequence was blasted on the EzTaxon-e server, after which the sequences of the nearest neighbors were retrieved and aligned, and neighbor-joining trees were built using the bootstrap test with 1000 replications.

### Living and dead cell preparation

Bacterial biomass was prepared by inoculating the 17OS isolate into a nutrient broth medium for 72 to 96 h at 35 °C with continuous agitation at a speed of 130 rpm. After incubation, live bacterial biomass was collected by centrifugation at 10,000 rpm at 4 °C for 10 min, followed by three washes with sterile saline water. Live bacterial biomass was collected, dried at room temperature inside a laminar air flow hood, and kept at 4 °C. For dead bacterial biomass, bacteria were autoclaved for 20 min at 121 °C and 15 lb pressure, centrifuged, and washed as previously mentioned. The dead bacterial biomass was dried in an oven at 60°C^27^.

### Optimization of Pb^2+^ metal removal by the 17OS strain

#### Minimum-run resolution IV design

Screening of the most significant variables affecting Pb^2+^ removal by *Paenibacillus dendritiformis* 17OS was studied by minimum-run resolution IV design. The statistical software package Design-Expert version 12 (Stat-Ease, Inc., Minneapolis, MN, USA) was applied to determine the relative importance of nutritional and environmental factors for Pb^2+^ removal by the selected *P. dendritiformis* 17OS. Six different variables, (Pb^2+^ concentration, cell type, pH, temperature, agitation speed, and incubation period) were selected to carry out this optimization process, as shown in Table 3. All trials proceeded in triplicate, and the design’s response was based on average results. Variables represented at two levels were Pb^2+^ concentration (200 and 500 mg/L), cell type (living and dead cells), and temperature (25 °C and 35 °C), whereas those represented at four levels were pH (5.5 and 7.0), agitation speed (0 and 150 rpm), and incubation period (12 and 24 h). Each row elucidated a trial run, and each column elucidated an independent variable.

The minimum-run resolution IV design was based on the first-order model, which was determined by the following equation (1):

1 $${\text{Y}} = {\text{ B}}_{0} + \Sigma {\text{B}}_{{\text{i}}} {\text{x}}_{{\text{i}}}$$

where Y is the response (metal removal), B_0_ is the model intercept, and B_i_ is the variable estimate.

Statistical analysis and graph plotting were performed using Design-Expert version 12. Analysis of variance (ANOVA) through Fisher’s test was used to detect the effect of independent variables on the response, and *p* < 0.05 identified significant results. Multiple determination coefficient (R^2^) and adjusted R^2^ were used as quality indicators to evaluate the fitness of the first-order equation. The standard error (SE) of the concentration effect was the square root of the variance of an effect, and the significance level (*p* value) of each concentration effect was determined using Student’s t-test t (Xi) in Eq. (2):

2 $${\text{t}}\left( {{\text{Xi}}} \right) = {\text{E}}\left( {{\text{Xi}}} \right)/{\text{SE}}$$

where E(Xi) is the variable Xi effect.

#### Box–Behnken design

A Box–Behnken design (CCD) was used to optimize the key variables after a minimum-run resolution IV design to identify the significant variables for Pb^2+^ removal by *P. dendritiformis* 17OS. The two levels (as low and high) of the four chosen independent variables were investigated, and batches of 29 tests (batch experiments) were carried out for the tested bacterium (Table 4).

The experimental data were analyzed using Design-Expert version 9.0.0. The independent variable values that produced the theoretical maximum response in Eq. (3) were optimal by maximizing the equation inside a specific boundary condition. Pb^2+^ elimination was identified as a response (Y), and data analysis using multiple regression techniques was performed to produce an empirical model linking the response measured to the independent factors. Using the second-order polynomial equation, the relationship between the independent variables and the outcome was determined [Eq. (3)].

3 $${\text{Y}}_{{\text{i}}} = {\text{b}}_{{0}} + {\text{b}}_{1} {\text{X}}_{1} + {\text{b}}_{2} {\text{X}}_{2} + {\text{b}}_{3} {\text{X}}_{3} + {\text{b}}_{11} {\text{X}}_{12} + {\text{b}}_{22} {\text{X}}_{22} + {\text{b}}_{33} {\text{X}}_{32} + {\text{b}}_{12} {\text{X}}_{1} {\text{X}}_{2} + {\text{b}}_{23} {\text{X}}_{2} {\text{X}}_{3} + {\text{b}}_{{1{3}}} {\text{X}}_{1} {\text{X}}_{3}$$

where Y_i_ is the predicted response, X_1_, X_2_, and X_3_ are independent variables, b_0_, is the offset term, b_1_, b_2_, b_3_ are linear effects, b_11_, b_22_, b_33_ are squared effects and b_12_, b_23_, and b_13_ are interaction terms.

Statistical analysis and graph plotting were performed using Design-Expert version 9.0.0. ANOVA through Fisher’s test was used to estimate the effect of independent variables on the response, and *p* < 0.05 identified significant results. Multiple determination coefficient (R^2^) and adjusted R^2^ were used as quality indicators to evaluate the fitness of the second-order polynomial equation. Contour plots (3D) and response surface curves were used to detect the relationship and interaction between the coded variables and the response. The optimal points were estimated by solving the equation derived from the final quadratic model.

### Preparation of the immobilized biosorbent/polyethersulfone (PES)

The most Pb^2+^-tolerant bacterium was encased in PES. The immobilization process was as follows: PES was first dissolved in an organic solvent at 20% concentration. The sonicated *P. dendritiformis* 17OS was added to the above-mentioned PES solution at 50% proportion and agitated for 180 min. The combined microbe solution was cast on a 300-mm-thick glass plate. These sheets were soaked in ultrapure water and washed. Finally, the immobilized biosorbents were used to adsorb Pb^2+^ from an aqueous solution.

#### Adsorption experiment

PES/biosorbents were introduced to a conical flask containing a solution of Pb ions and stirred at 150 mg/L. Pb(NO_3_)_2_ was used to make the Pb solution. The dry weight of biosorbents was 0.5, 1, 1.5, and 2 g/L. Moreover, neat PES with the same dry weight was used as a control. The starting Pb(II) concentration in the adsorption trials was 200 mg/L.

The biosorption of Pb (%) was calculated by the equation of Shetty and Rajkumar^28^:

4 $${\text{Efficiency of biosorption}}\left( \% \right) = \frac{CI - CF}{{CI}} \times 100$$

where CI is the initial metal concentration and CF is the final metal concentration (residual).

#### Physicochemical characteristics of biosorbents

The morphological and structural images of pure PES membrane and immobilized biosorbents were analyzed by a Quanta FEG-250 microscope at a voltage of 20 kV after gold plating at an accelerating voltage.

The functional groups of biosorbents were identified using FTIR spectroscopy (JASCO FTIR 4100 spectrometer, Japan) at the National Research Center (Doki, Egypt). The frequency range used to scan the spectra was 400 to 4000 cm^−1^ (with a resolution of 4 cm^−1^ and 60 scans).

The water contact angle was the method to examine the hydrophilicity of the membrane. The contact angle values were obtained by (SCA 20, OCA 15 EC) using the sessile drop method. The volume and contact time were10μL and 10 s, respectively, with five times for each membrane.

Zeta potential (ζ) was determined through dynamic and electrophoretic light scattering (Particle Sizing Systems, Inc.Santa Barbara, Calif., USA). 0.05 g of membrane-making powder was dispersed in 5 mL solvent.

#### Statistical analysis

Data were statistically determined using the IBM^®^ SPSS^®^ Statistics software version 19 on the premise of Duncan’s multiple range test at the 5% level^29^. All analyses were performed in triplicate.

## Results and discussion

### Isolation and screening of Pb-tolerant bacteria

On agar medium containing 1000 mg/L Pb (NO3)2﻿, 41 bacterial isolates were isolated from various effluent and contaminated soil samples obtained from EF, OS, GS, and SW sites.

**Figure 2:**
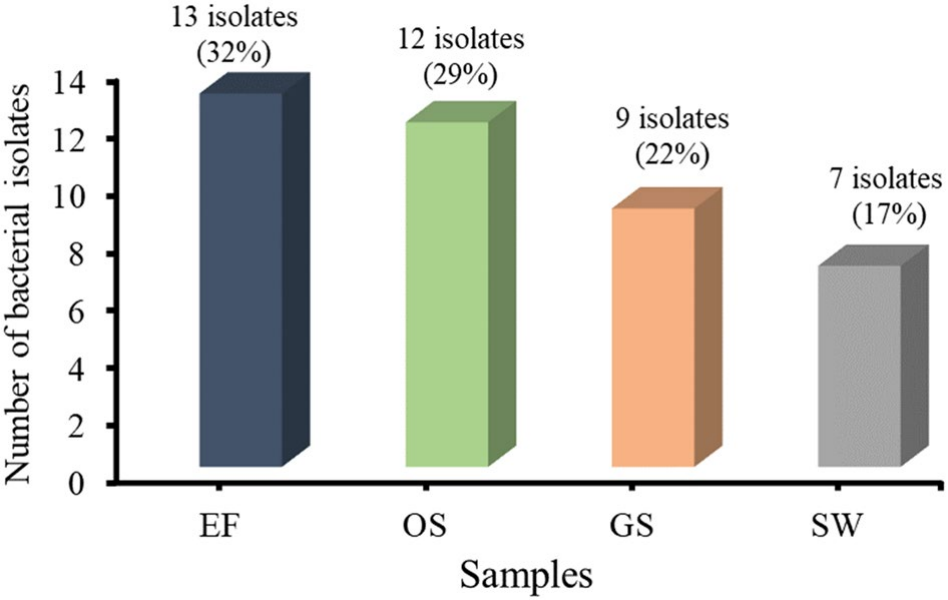
Number and percentage distribution of lead-tolerant bacteria from different contaminated sites. EF*: Electrical factory, OS: oil and soap company, GS: Gas station, and SW: soil near sewage water.

Figure 2. demonstrates the distribution number and percentage of bacterial isolates collected from various sources after 48 h incubation at 30 °C. The EF effluent included the greatest number of Pb^2+^-tolerant bacterial isolates (13 isolates, representing 32% of the total), whereas 12, 9, and 7 bacterial isolates (representing 29%, 22%, and 17%, respectively) were isolated from the soil of OS, GS, and SW, respectively.

A total of 41 isolates grew on the Pb^2+^ metal at a high concentration of 1000 mg/L and could be categorized into four groups (weak, moderate, high, and very high) based on the agar medium growth degree (ranging from+ to +  +  + +), as shown in Table 2. Results indicated that two bacterial isolates of the 8EF and 17OS codes had very high growth efficiency (+ +  + +) on the Pb^2+^ metal, ranking them in the fourth group. Furthermore, 10 isolates with high growth (+ + +) efficiency and 7EF, 11EF, 18OS, 19OS, 22OS, 26GS, 28GS, 34GS, 36SW, and 39SW codes were placed in the third group, whereas 14 and 15 isolates showed low ( +) and intermediate (+ +) growth efficiency on Pb^2+^ and were rated first and second, respectively. As a result, the most efficient Pb^2+^ metal-tolerant isolates (8EF and 17OS) were chosen for the following trial.

**Table 2:** Growth efficiency of bacterial isolates on solid media supplemented with Pb^2+^ heavy metal.

Isolation source	Isolate code	Growth degree	Isolation source	Isolate code	Growth degree	Isolation source	Isolate code	Growth degree
EF	1EF	+	OS	15OS	++	GS	29GS	++
	2EF	++		16OS	++		30GS	++
	3EF	+		17OS	++++		31GS	+
	4EF	++		18OS	+++		32GS	+
	5EF	++		19OS	+++		33GS	++
	6EF	+		20OS	+		34GS	++ +
	7EF	+++		21OS	+	SW	35SW	++
	8EF	++++		22OS	+ ++		36SW	+ + +
	9 EF	++		23OS	++		37SW	++
	10EF	+		24OS	++		38SW	++
	11EF	+++		25OS	+		39SW	+++
	12EF	++	GS	26GS	+++		40SW	+
	13EF	+		27GS	+		41SW	+
OS	14OS	+		28GS	+++			

EF* Electrical factory, *OS* Oil and soap company, *GS* Gas station, and *SW* Soil near sewage water. + , Weak growth; ++,  Moderate growth; +++, High growth; ++++, Very high growth.

These results agree with Helmy et al.^30^ who found that 100 of the 123 bacterial isolates showed growth on agar plates with heavy metal ions, and their positive findings ranged from (+) to (+ +  + +) depending on the growth density, which ranged from very low to high. Moreover, Abd El Hameed et al.^31^ revealed that out of 26 fungal isolates,18 isolates showed growth on agar plates amended with metal ions and showed positive findings ( +).

### Evaluation of MIC, MTC, and multimetal resistance for the selected tolerance Pb^2+^ isolates

**Figure 3:**
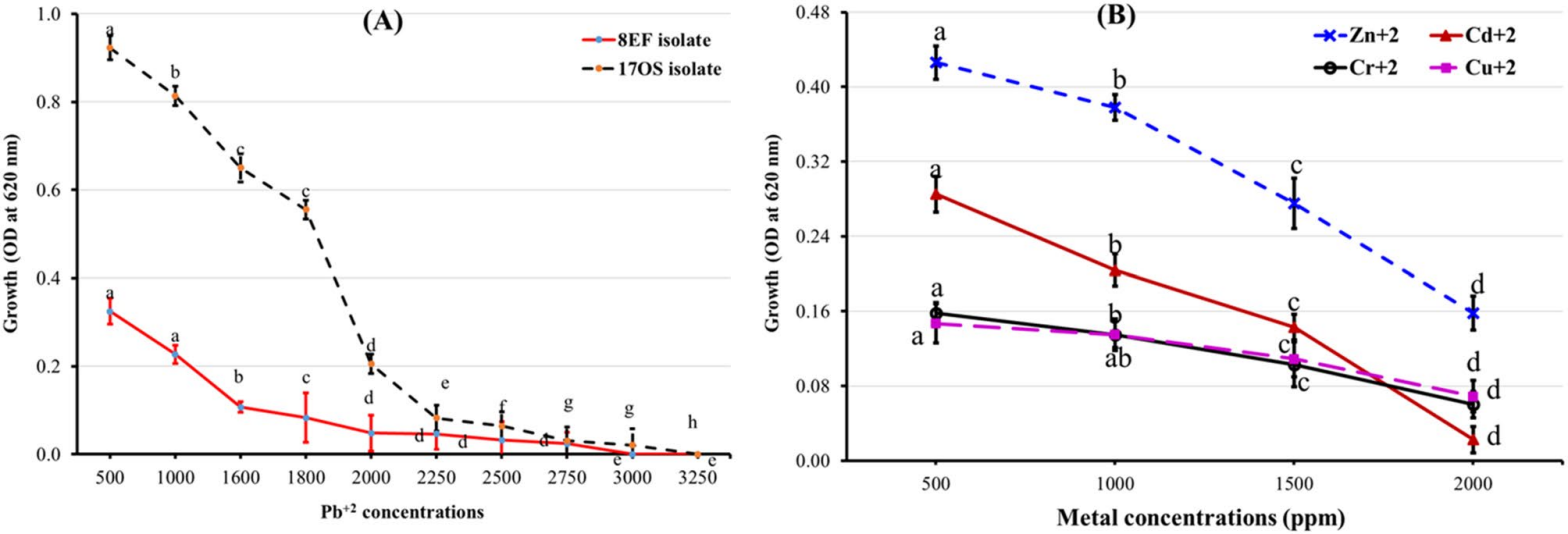
Growth density (O.D) of both 8EF and 17OS isolates as affected by lead concentrations (**A**) and 17OS isolate as influenced by multi-metal (Zn^+2^, Cd^+2^, Cr^+6^, and Cu^+2^) concentrations of (**B**). ^a,b^Values with small letters above the same line having different superscripts are the significant difference (at *p* ≤ 0.05). Standard deviation bar.

The selected isolates 8EF and 17OS were grown on a broth medium containing Pb^2+^ metal at concentrations ranging from 500 to 3250 mg/L. Figure 3A shows the ability of the selected isolates 8EF and 17OS to tolerate different Pb^2+^ metal concentrations for a 48 h incubation period, and the growth (expressed as O.D) ranged from 0.325 to 0.024 and 0.923 to 0.021, respectively. The 17OS isolate appeared with the MTC at 3000 mg/L with a MIC value of 3250 mg/L, whereas the 8EF isolate achieved MTC at 2750 mg/L with a MIC value of 3000 mg/L.

Similar results were revealed by Mohapatra et al.^32^, who showed that the PbRPSD202 isolate recorded high tolerance to Pb(II) (2150 mg/L) with a MIC value of 2200 mg/L. In contrast, Abd El Hameed et al.^31^ recorded that the tested fungal isolates could grow on a broth medium supplemented with different Pb^2+^ concentrations and a MTC at 150 mg/L. Moreover, El-Meihy et al.^33^ observed that the three isolates of UR25, UR27, and MR98 grew on a medium supplemented with Cd^2+^ at a MTC of 1500 mg/L and inhibited at a MIC value of 2000 m/L, whereas MR99, MR100, and MR108 isolates grew with an MTC of 2000 mg/L Cd^2+^ and inhibited at a MIC value of 2500 mg/L. From these results, the 17OS isolate was selected as the best isolate for further study, which exhibited a high tolerance of Pb^2+^ metal at a high concentration. Sanket et al.^34^ suggested that native bacteria undergo several mechanisms for tolerating lead concentrations. These mechanisms include effluxing of metal, enzymatic conversion, sensitivity reduction of cellular targets, permeability barrier exclusion, and cellular sequestration. In addition, Das et al.^35^ found that Bacterial strains may acquire a resistance system against metal toxicity as a result of repeated exposure to metal contaminants.

Because understanding strain tolerance to various heavy metals is a requirement for investigating biosorption, the selected isolate 17OS was evaluated for multimetal (Zn^2+^, Cd^2+^, Cr^6+^, and Cu^2+^) resistance at different concentrations of tested heavy metals (Fig. 3B). Results clearly showed that this isolate recorded O.D (growth) ranging from 0.125 to 0.426, 0.0225 to 0.285, 0.06 to 0.185, and 0.069 to 0.147 in a broth medium supplemented with concentrations from 500 to 2000 mg/L of Zn^2+^, Cd^2+^, Cr^6+^, and Cu^2+^, respectively. Thus, the 17OS isolate tolerated multi-metals at a high concentration, reaching 2000 mg/L. In this respect, Helmy et al.^30^ demonstrated that the bacterial isolates tolerated high concentrations of various heavy metals (Al^3+^, Zn^2+^, Cr^5+^, and Ni^2+^) up to 17.76, 224.03, 70.4, and 1952 mg/L, respectively. Mohapatra et al.^32^ also found that *Bacillus xiamenensis* is tolerant of high Cd(II), Cr(VI), As(III), Ni(II), Cu(II), and Zn (II) concentrations up to 500, 3000, 150, 100, 150, and 50 mg/L, respectively.

Results suggested that low Pb^2+^ concentrations (500 mg/L) stimulate bacterial growth, which may be related to heavy metal uptake by cells. The cell initially accumulates metal, and subsequent mineralization is carried out to create nontoxic metals^36^. Metals, such as copper and zinc, are essential for bacteria because they offer necessary cofactors for some proteins and enzymes at relatively low concentrations^37^. However, the high concentration of other metals, such as Pb^2+^, can seriously impede bacterial growth. Hu et al.^38^ obtained similar results.

### Identification of the most efficient isolate (17OS)

**Figure 4:**
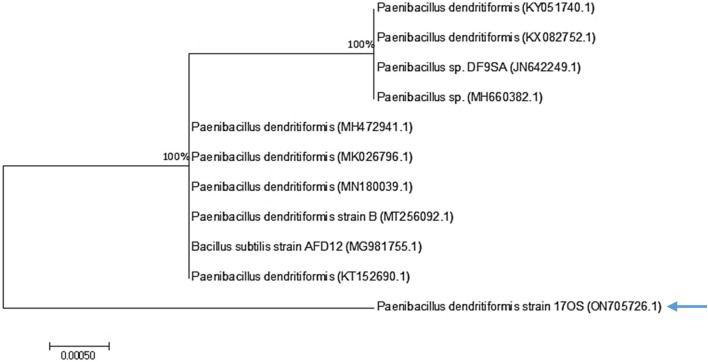
Phylogenetic tree constructed using 16S rDNA sequences of 17OS isolate using the neighbor-joining method (Evolutionary analyses were conducted using MEGA7).

The most potent isolate (17OS) was identified up to the genus according to phenotypic (cultural, morphological, and physicochemical) characteristics^24^. This isolate was classified as *Paenibacillus*, which appeared as rod-shaped, Gram-positive, motile, and aerobic; gave positive reaction of catalase, lipase, and amylase; and grew in temperatures ranging from 5 to 55 °C and pH levels ranging from 7 to 9.5 and in the presence of 2 to 6% NaCl. In addition, this genus was confirmed by molecular identification using 16S rRNA as *P. dendritiformis* 17OS with 100% similarity and deposited in GenBank under accession number ON705726.1. Figure 4. shows a phylogenetic tree between the selected strain and other strains. In this regard, Sridevi and Raghuram^39^ isolated *P. dendritiformis* from contaminated soil and deposited it in GenBank under accession number MK100387. This isolate exhibited high tolerance against three metals (Pb > Zn > Cu) at its optimum pH. Additionally, microorganisms such as *Bacillus* sp. PZ-1 and *Pseudomonas* sp. 13 have been found to adsorb Pb (II) from wastewater^40^.

### Optimization of Pb^2+^ metal removal by the ***P. dendritiformis*** 17OS strain

#### Minimum-run resolution IV design

The most significant factors were screened using the minimum-run resolution IV experimental design matrix. Table 3 shows a wide variation in the value of Pb^2+^ metal removal by 17OS strain that ranged from 198.2 to 498.6 mg/L in a 14-run trial. The maximal Pb^2+^ metal removal value (498.6 mg/L) was achieved in a run number of 11 with low levels of pH (5.5), dead cells (6.8 g/L), and incubation period (24 h) and high levels of temperature degree (35 °C), metal concentration (500 mg/L), and agitation speed (150 rpm), whereas the lowest Pb^2+^ metal removal value (198.2 mg/L) was recorded in a run number of 14 with low levels of temperature degree (25 °C), metal concentration (200 mg/L), and dead cells (6.8 g/L) and high levels of pH (7), incubation period (48 h), and agitation speed (150 rpm), respectively. The impact of independent factors on the response was assessed using ANOVA through Fisher’s test, and significant results were indicated by *p* < 0.05. The 17OS isolate’s ability to remove Pb^2+^ metal is demonstrated by the model’s *F*-value of 2697.84, as listed in Table 3. Generally, a high significance of the associated coefficient is indicated by a bigger F-value and a smaller *p* value^41^. The factor with the highest F-value is regarded as the best and is given the highest ranking. The factors were rated in the following order based on F-values (Table 3): metal concentration, agitation speed, temperature level, cells, and pH. A big coefficient for a variable (positive or negative) indicates a significant influence on the outcome. A positive sign of the tested variable’s effect denotes a higher level of the variable’s influence on removal, whereas a negative sign denotes a lower degree of the variable’s influence. Analysis of the regression coefficients of six factors confirmed that metal concentration and temperature degree had a positive effect on Pb^2+^ removal, whereas agitation speed, incubation period, cells, and pH had a negative effect on Pb^2+^ removal. The corresponding probability values (*p* values) indicate the significance of each coefficient. Table 3 shows that four factors are significant (*p* < 0.001), proposing the significance of the model were temperature degree (*p* = 0.032), metal concentration (*p* = 0.005), incubation period (*p* = 0.027), and agitation speed (*p* = 0.027). The SE of all variables was 1.08.

**Table 3:** Minimum Run Resolution IV experimental design matrix, the results of actual values, analysis of variance (ANOVA), and fit statistics of variables affecting Pb^2+^ metal removal by 17OS strain.

Run no.		Temperature (°C)	pH	Metal concentrations (mg/L)	Cells (g/L)	Incubation periods (h)	Agitation speed (rpm)	Removal heavy metals (mg/L)
Levels Low		25	5.5	200	1 = (6.8 g/L) dead cells	12	0	
High		35	7.0	500	2 = (6.8 g/L) living cells	24	150	
1		35	5.5	200	1	48	0	199.4
2		25	5.5	200	2	24	0	198.3
3		35	7	500	2	48	0	494.5
4		25	5.5	500	1	48	0	496.9
5		35	5.5	500	2	24	0	498.3
6		35	5.5	500	2	48	150	493.8
7		25	7	200	1	24	0	199.4
8		35	7	200	2	24	150	198.4
9		25	5.5	200	1	24	150	199.5
10		35	7	500	1	48	150	494.4
11		35	5.5	500	1	24	150	498.6
12		25	7	500	2	24	150	492.2
13		25	7	200	2	48	0	199.3
14		25	7	200	1	48	150	198.2

Analysis of variance (ANOVA)								
	Model							
Mean^2^	33,723.04	4893.6	3.53	1.77E + 05	10.67	6777.79	7049.34	
F value	2697.84	391.49	0.2821	14,175.28	0.8533	542.22	563.95	
*p* value	0.015*	0.032*	0.689	0.005*	0.525	0.027*	0.027*	
Effect		49.47	− 1.33	297.65	− 2.31	− 58.21	− 59.37	
CE	322.27	24.73	− 0.575	148.82	− 1	− 25.21	− 25.71	
SE	1.08	1.08	1.08	1.08	1.08	1.08	1.08	

Fit statistics								
SD		3.54		R-squared		1.00		
Mean		332.73		Adj R-squared		0.99		
Adeq precision		146.19		Pred R-squared		0.96		

*Mean*^2^ Mean Square, *P* Corresponding level of significance, *F* Corresponding level of significance, *CE* Coefficient estimate, *SE* Standard error, *SD* Standard deviation, *R*2 Determination coefficient, *Adj*. Adjusted, *Pred*. Predicted, *Adeq*. Adequate.

*Significant at 5% level (*p* < 0.05).

Statistical analysis (Table 3) showed that the standard deviation and mean were 3.54 and 332.73, respectively. Adequate precision measures the signal-to-noise ratio, and the ratio was 146.19, which was > 4; it was desirable and indicated an adequate signal. Data also indicated that the R^2^ was high determination (1.00), which means that the model explained 100% of the total variation, and the predicted R^2^ of 0.96 was in reasonable agreement with the adjusted R^2^ of 0.99. Therefore, the actual values were compatible with the predicted values, suggesting that the data matched the model well (Fig. S1).

Regression analysis was performed on the results, and the first-order polynomial equation was derived [Eq. (5)].

5 $$\begin{aligned} {\text{Y}} & = {322}.{27} + ({24}.{73}*\;{\text{temperature degree}}) + ( - 0.{575}*\;{\text{pH}}) + ({148}.{82}*\;{\text{metal concentration}}) + \left( { - { 1}*\;{\text{cells}}} \right) \\ \qquad + & ( - {25}.{21}*\;{\text{incubation periods}}) + ( - {25}.{71}*\;{\text{agitation speed}}) \\ \end{aligned}$$

The one-factor and interaction between two-factor plots were also systematically estimated in an optimal custom design for the best biomass production demonstrated through models in Supplementary Figs. S2 and S3. The interaction between factors revealed by two nonparallel lines occurred when another influenced one factor. While not interacting, the factors were presented in parallel lines.

In general, biosorption is an exothermic process; hence, metal adsorption on biomass decreases as temperature increases^42^. Dharanguttikar^43^; Wang and Chen^44^ reported that the change in temperature influences several factors, such as the stability of metal ions in the solution, cell wall configuration of microorganisms, and ionization energy of the metal–biomass complex. In addition, Wang and Chen^44^ studied dead and living biomass biosorption capacity. This study showed that dead biosorbents exhibit higher biosorption efficiencies or capabilities than the corresponding living cells at selected key experimental parameters.

#### Box–Behnken design

The most significant factors (temperature degree, metal concentration, incubation period, and agitation speed) were selected from a minimum-run resolution IV experimental design and maximized the Pb^2+^ metal removal by 17OS strain through response surface methodology using the Box–Behnken design. The design matrix of the tested variables was based on 29 experimental runs and the experimental results (Table 4). Results showed that trial run number 22 increased the metal removal to 500 mg/L with combinations of temperature (40 °C), metal concentration (600 mg/L), incubation period (18 h), and agitation speed (150 rpm), whereas the lowest metal removal achieved in trial run number 2 was 395 mg/L with combinations of temperature (37.5 °C), metal concentration (500 mg/L), the incubation period (12 h), and agitation speed (125 rpm).

**Table 4:** Box–Behnken design matrix and the actual values, analysis of variance (ANOVA), and fit statistics of variables affecting  lead metal removal by dead biomass of 17OS strain.

Run no.		A	B	C	D		Pb^2+^ remove (mg/L)	
1		37.5	700	12	125		477.0	
2		37.5	500	12	125		395.0	
3		35.0	600	12	125		442.0	
4		35.0	600	18	150		492.1	
5		37.5	700	24	125		411.0	
6		35.0	500	18	125		445.2	
7		37.5	500	18	150		476.4	
8		37.5	600	18	125		460.0	
9		37.5	600	18	125		468.5	
10		37.5	500	24	125		458.0	
11		37.5	600	12	150		467.7	
12		40.0	600	18	100		470.0	
13		37.5	600	18	125		458.3	
14		37.5	600	12	100		469.3	
15		35.0	700	18	125		467.6	
16		35.0	600	18	100		466.7	
17		37.5	600	18	125		469.3	
18		35.0	600	24	125		483.0	
19		40.0	500	18	125		465.2	
20		40.0	600	24	125		457.7	
21		37.5	600	24	100		447.9	
22		40.0	600	18	150		520.0	
23		37.5	600	18	125		469.2	
24		37.5	700	18	100		468.7	
25		37.5	500	18	100		425.0	
26		40.0	600	12	125		476.0	
27		40.0	700	18	125		460.0	
28		37.5	700	18	150		468.0	
29		37.5	600	24	150		488.1	

Analysis of variance (ANOVA)								
	Model	A	B	C	D	AB	AC	AD
Mean Square	965.34	86.94	638.02	29.14	1744.84	190.44	879.12	5.29
F value	31.95	2.88	21.12	0.9645	57.75	6.30	29.10	0.18
*p* value	0.0001*	0.1119	0.0004*	0.3427	0.0001*	0.025*	0.0001*	0.682
CE	465.06	2.69	7.29	1.56	12.06	− 6.9	− 14.83	1.15
SE	2.46	1.59	1.59	1.59	1.59	2.75	2.75	2.75

	BC	BD	CD	A^2^	B^2^	C^2^	D^2^	Lack of fit
Mean Square	4160.25	678.6	436.81	536.06	1865.6	649.84	769.36	30.47
F value	137.69	22.46	14.46	17.74	61.75	21.51	25.46	1.03
*p* value	0.0001*	0.0003*	0.0019*	0.0009*	0.0001*	0.0004*	0.0002*	0.53
CE	− 32.25	− 13.02	10.45	9.09	− 16.96	− 10.01	10.89	
SE	2.75	2.75	2.75	2.16	2.16	2.16	2.16	

Fit statistics								
SD		5.5		R-Squared		0.97		
Mean		462.17		Adj R-Squared		0.94		
Adeq Precision		26.30		Pred R-Squared		0.86		

*A* Temperature degree, *B* Metal concentration, *C* Incubation periods and *D* Agitation speed. Mean^2^ = Mean Square *P* corresponding level of significance, *F* corresponding level of significance, *CE* Coefficient Estimate, *SE* Standard Error, *SD* Standard Deviation, *R*2 Determination coefficient, *Adj*. Adjusted, *Pred*. Predicted, *Adeq*. Adequate.

*Significant at 5% level (*p* < 0.05).

Table 4 reveals that the model *F*-value of 31.95 implies that the model is significant (*p* < 0.0001) for Pb^2+^ metal removal by the 17OS strain. Individual terms of metal concentration (B) and agitation speed (D) and interaction terms of AB, AC, BC, BD, and CD were significant model terms (*p* < 0.05). Moreover, quadratic A^2^, B^2^, C^2^, and D^2^ were significant. The standard deviation and mean were 5.5 and 462.17. Adequate precision measures the signal-to-noise ratio. The ratio was 26.30, which was more significant than 4; it was desirable and indicated an adequate signal. Data also indicated that the R^2^ was high determination (0.97), which means that the model explained 97% of the total variation, and the predicted R^2^ of 0.86 was reasonable with the adjusted R^2^ of 0.94 agreement (high correlation; Fig. S4). The difference was < 0.2. The adjusted determination coefficient refers to the proportion of the variation in the response explained by the regression model. Data were analyzed using multiple regression analysis to get an empirical model for the best response and derive a second-order polynomial equation [Eq. (6)].

6 $$\begin{aligned} {\text{Y}} & = {465}.0{6} + ({2}.{69}*{\text{A}}) + ({7}.{29}*{\text{B}}) + ({1}.{56}*{\text{C}}) + ({12}.0{6}*{\text{D}}) + ( - {6}.{9}*{\text{AB}}) + ( - {14}.{83}*{\text{AC}}) + ({1}.{15}*{\text{AD}}) \\ \qquad + & ( - {32}.{25}*{\text{ BC}}) + ( - {13}.0{2}*{\text{ BD}}) + ({1}0.{45}*{\text{CD}}) + ({9}.0{9}*{\text{A}}^{{2}} ) + ( - {16}.{96}*{\text{B}}^{{2}} ) + ( - {1}0.0{1}*{\text{C}}^{{2}} ) + ({1}0.{89}*{\text{D}}^{{2}} ) \\ \end{aligned}$$

where *A* Temperature degree, *B* Metal concentration, *C* Incubation periods, and *D* Agitation speed.

**Figure 5:**
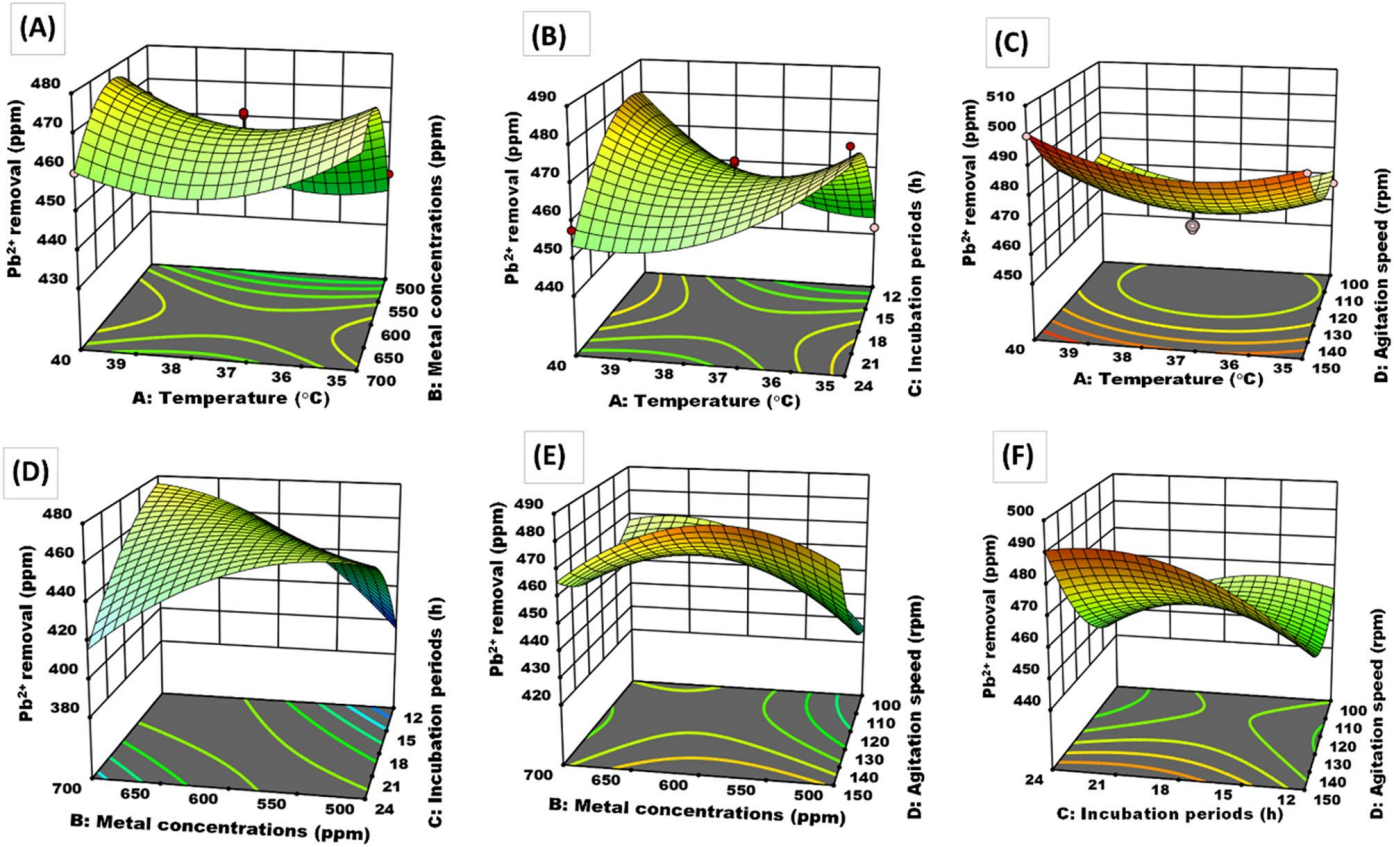
Three-dimensional response surface and two-dimensional contour plots Pb^2+^ metal removal by 17OS strain showing variable interactions of (**A**) Temperature degree vs. metal concentration, (**B**) Temperature degree vs. incubation periods, (**C**) Temperature degree vs. agitation speed, (**D**) metal concentration vs. incubation periods, (**E**) metal concentration vs. agitation speed and (**F**) incubation periods vs. agitation speed.

Three-dimensional response surface and two-dimensional contour plots were graphically based on the model equation to explain the interaction among variables and determine each factor’s optimum level for Pb^2+^ metal removal by 17OS strain, as shown in Fig. 5A–F. Figure 5A shows the response surface plot based on independent variables (i.e., temperature degree and metal concentration), with the other independent variable kept at zero. Figure 5A reveals an interaction behavior with a negative main effect (− 6.90) of temperature degree and metal concentration, indicating that a decrease in temperature degree and metal concentration values yielded high Pb^2+^ removal. A similar response curve in Fig. 5B shows that the interaction between temperature degree and incubation period with a negative main effect (− 14.83) and the other independent variable was kept at zero level to achieve a maximum Pb^2+^ removal. In Fig. 5C, the interaction between temperature degree and agitation speed with a positive main effect (1.15) and the other independent variable was kept at the zero level. The maximum Pb^2+^ removal was attained with a positive main effect of temperature degree and agitation speed at high levels. Metal concentration was also involved in a two-way interaction with the incubation period (Fig. 5D), but its main effect remained negative (− 32.25). Figure 5D shows the 3D curve, with a negative main effect of metal concentration and incubation period with the other independent variable kept at the zero level.

Moreover, Fig. 5E shows that the interaction with a negative main effect (− 13.02) was recorded when the other independent variable was kept at zero. In addition, Fig. 5F shows that the interaction between the incubation period and agitation speed was a positive main effect (10.45), and both variables were positive main effects. The highest removal was achieved at high levels of incubation period and agitation speed.

These results agree with Aslam et al.^45^ who reported that the percentage of lead accumulation by *Stenotrophomonas* sp. MB339 rapidly decreased with increasing temperature up to 45 °C that's because the temperature is known to alter the stability of the cell wall and its configuration. On the contrary, Banerjee et al.^46^ suggested that higher temperatures often increase metabolic activity and system energy, which would contribute to the active uptake of metals. In addition, Ozdemir et al.^47^ showed that biosorption of metals is an energy-independent mechanism, thus the temperature of the biosorption was less significant in comparison to the effect of other physicochemical factors.

### Validation of the model

Many validation trials were carried out in the experimental area bound by the factorial points (xi ranging between − 1 and + 1) to examine the prediction power of the constructed model. The RSM tool's unique function, 'Point prediction,' was used to determine the optimal value of the combination of the four parameters for maximal metal removal. The actual values of Pb^2+^ metal removal by 17OS strain (520.00 mg/L) were in good agreement with the predicted values (512.61 mg/L) and were within the 95% confidence prediction intervals, further confirming the model presented above. The predicted ideal conditions were found: temperature, 40 °C; metal concentration, 600 mg/L; incubation periods, 18 h; and agitation speed, 150 rpm. Furthermore, the sequential optimization technique was interested in increasing Pb^2+^ removal by 4.29% using the Box–Behnken design when compared to the minimum run resolution IV experimental design. Furthermore, the Box–Behnken design (BBD), a Response Surface Method (RSM), was used to optimize the biosorption of Pb metal. The six major factors; pH, biosorbent dosage, and metal concentration, were optimized to remove the metals efficiently^48^.

### Effect of biosorbent dosage on adsorption performance

**Figure 6:**
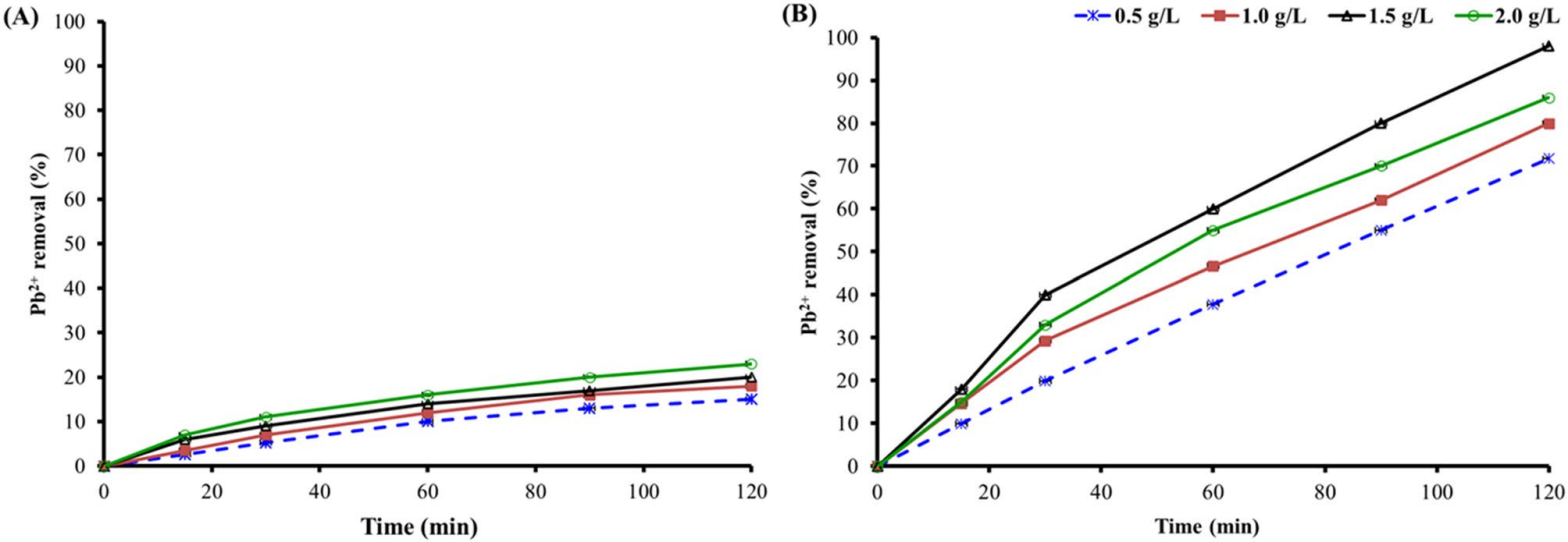
Impact of loading dose for adsorption of Pb^2+^ ions. (**A**) neat PES (**B**) PES/biosorbents. Satandard division bar.

PES possesses good chemical and mechanical properties; thus, it is used in membrane fabrication for water treatment^49^. The amount of Pb^2+^ ions eliminated was considerably impacted by the quantity of biosorbent employed^50^. In this experiment, the starting Pb^2+^ ion concentration was 200 mg/L, and the dry weight of the biosorbents or neat PES was 0.5 to 2 g/L. The removal level of Pb^2+^ ions from 0.5 to 2 g for neat PES was about the same as the lowest removal rate and reached only 23% (Fig. 6A). In contrast, as the dosage of the biosorbent was increased, the rate of Pb^2+^ ion adsorption increased, as shown in Fig. 6B. When the biosorbent dosage was increased from 0.5 to 1.5 g/L, the elimination rate rose from 72 to 98%. This occurrence was attributed to increased adsorption sites on the biosorbent’s surface^51^. The removal rate of Pb ions was also boosted by the adsorption dynamic generated by varied concentration gradients.

However, when the dosage of the biosorbent was increased to 2 g/L, the removal rate of Pb ions decreased to 85%, as shown in Fig. 6B, because by increasing the biosorbent dosage, the adsorption agent reunion overlapped, decreasing the number of accessible adsorption sites and lowering the removal rate^52^. Another explanation was that the biosorbent dosage was steadily increased, whereas the number of biosorbent sites could not attain saturation. The biosorbent utilization coefficient dropped. The removal rate was also reduced due to the repulsive interactions between the adsorption sites and the increased electrostatic interaction^53^. Thus, the removal of Pb^2+^ ions was significantly improved when *P. dendritiformis* 17OS was used as a biosorbent.

### Characterization of biosorbent

#### SEM observation

**Figure 7:**
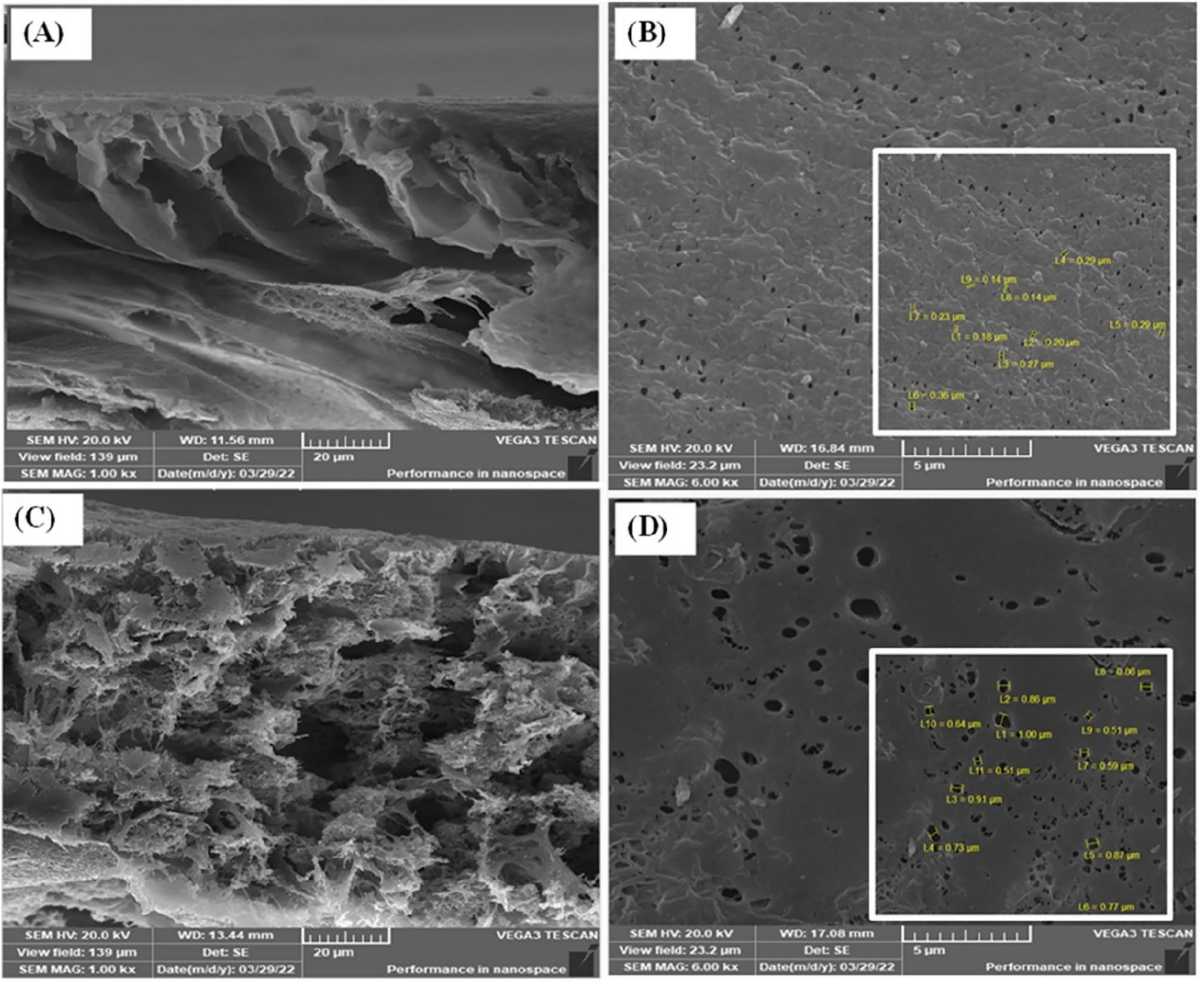
SEM images for the prepared biosorbents, (**A**) The internal structure of the blank PES, (**B**) The surface structure of the PES, (**C**) The internal structure of the immobilized biosorbent, and (**D**) The surface structure of the immobilized biosorbent.

Figure 7A–D shows the cross-sections of pure PES membranes and PES/biosorbents. The cross-section of neat PES has a fingerlike shape. Bacteria are uniformly dispersed in the polymeric matrix over the entire membrane, resulting in larger pores than pure PES membranes. Including *P. dendritiformis* 17OS into the PES dope accelerates the phase inversion, resulting in larger holes developing in PES/biosorbents. Furthermore, compared to pure PES membranes, the skin layers on the top of PES/biosorbents are smoother. The structure of PES/biosorbents results in a greater adsorption capacity compared to a pure PES membrane. From the inset of the surface images, the pore size of the modified PES membranes with bacterial cells was increased from 0.21 to 0.78 µm.

#### FTIR analysis

**Figure 8:**
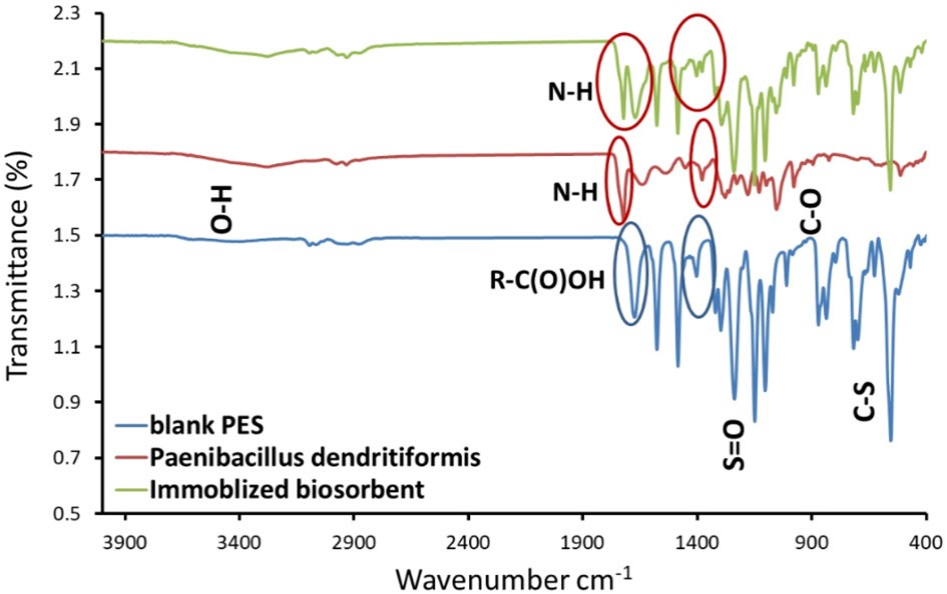
FT-IR spectrum of neat PES, *Paenibacillus dendritiformis* 17OS, and immobilized biosorbents.

The biosorbents were analyzed by FTIR spectroscopy, as shown in Fig. 8. The characteristic peaks were found in 1196, 1495, and 1814 cm^−1^, and this phenomenon elucidated that the surface had the functional groups, such as P–O, –COOH, and C=O, after immobilization^54^. For the blank PES, the characteristic band for the sulfonation group was observed at 1141 and 1244 cm^−1^. Moreover, the peak at 1657 cm^−1^ was owned to aromatic in-plane ring bend stretching vibration for the blank PES.

#### Estimation of hydrophilic properties

The water contact angle is a test to confirm the hydrophilic nature of the immobilized membrane. As shown in Table 5. The immobilized PES membrane surface properties were modified and become more hydrophilic. The biosorbents were capable of reducing the membrane hydrophobicity of the neat PES. The contact angle decreased from 61 degrees for PES to 30.4 for the immobilized. This is owed to the composition of the bio sorbents and the presence of the functional groups, such as P–O, –COOH, C=O –OH and –NH on the surface.

**Table 5:** Contact angle of naet PES and PES/biosorbent.

Membranes	Contact angle	
neat PES		61 degree
PES/biosorbents		30.4 degree

The surface ζ values of the solution neat PES was 4.2 mV and the solution composition for PES/biosorbents had a negative outer surface f value (− 9.10 mV) due to the carboxyl acid groups of biosorbents. Obviously, by loading the biosorbents with PES solution, a high negative ζ value was attained. These results confirm that the negatively charged characteristics of immobilized membranes were enhanced with the incorporation of biosorbents in the PES solution. So the mechanism of adsorption is attributed to the electrostatic attraction (physico-sorption) between PES/biosorbents membranes and lead ions.

#### Adsorption isotherms

**Figure 9:**
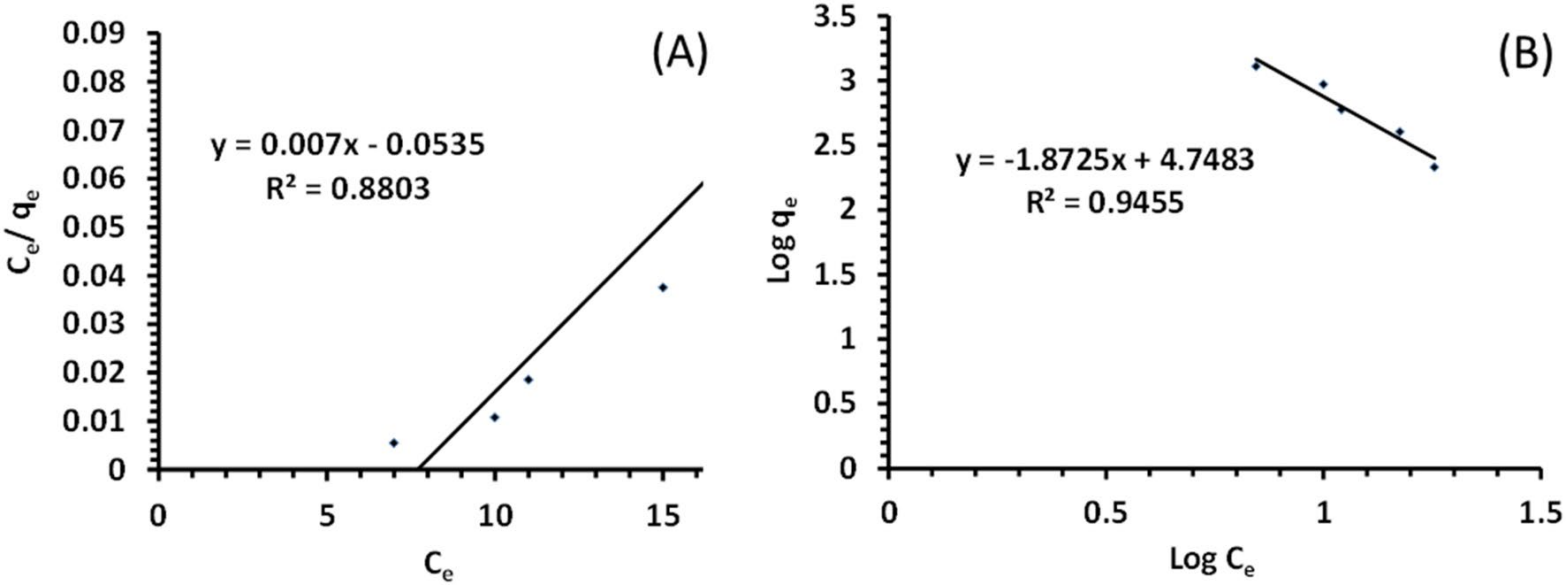
Adsorption isotherm models (**A**) Plot of qe and Ce for linear Langmuir (**B**) Plot of log qe and log Ce for linear Freundlich.

The Pb(II) ions adsorption isotherms and the Pb(II) adsorption capacity for PES/biosorbent, are shown in Fig. 9. Two of the adsorption isotherms models were used to describe the interactive behavior between adsorbents and heavy metals. The interaction behavior between adsorbent and adsorbate can be estimated by applying the well-established fundamental Langmuir and Freundlich isotherm models. The Langmuir isotherm model represents monolayer adsorption, and the Freundlich isotherm model represents multilayer adsorption. The PES/biosorbent showed a maximum adsorption capacity of 144 mg g^−1^ for Pb(II) at neutral pH. It demonstrates that the PES/biosorbent has shown considerably higher Pb(II) ions adsorption capacity at neutral pH. Adsorption isotherms Pb(II) was well known, Langmuir (Eq. 7) and Freundlich (Eq. 8) isotherm models are used to obtaining the adsorption equilibriums data, which can be expressed as follows.

7 $$C_{e} /q_{e} = \left( {1/q_{max} } \right)b + \left( {C_{e} /q_{max} } \right)$$

where C_e_ is the concentration (mg/L) at equilibrium, q_e_ is the quantity of adsorbed lead ions (mg/g) at equilibrium, $q_{max}$ (mg /g) and b (L/ mg) are the Langmuir constants which are identified with the adsorption limit and adsorption energy respectively.

8 $${\text{Log}} q_{e} = \log K_{F} + \frac{1}{n}{\text{ln}}C_{e}$$

where K_F_ is steady and characterized as the adsorption or distribution identified with the bonding energy, it explains the amount of ions adsorbed on the adsorbent surface (mg /g) which is a proportion of adsorption limit. A plot of ln q_e_ versus ln C_e_ allowed a straight line with a slope equal (1/n) and intercept equal ln K_F_.

The values indicate that the Freundlich model has a good fitting and shows a higher regression coefficient value than the Langmuir model. Pb(II) adsorption on PES/biosorbent has better fitting by the Freundlich isotherm model, indicating a multilayer adsorption mechanism.

## Conclusions

In summary, *P. dendritiformis* 17OS was isolated from an OS contaminated site and could tolerate a high Pb^2+^ metal concentration in the growth medium. It can also be resistant to multimetals at high concentrations. The ability of this strain to Pb^2+^ metal removal was improved after optimizing the growth parameter of temperature degree, pH level, Pb^2+^ concentration, cell type, agitation speed, and incubation period using a statistical design experiment, where the Pb^2+^ metal removal was increased by ~ 4.29%. *P. dendritiformis* 17OS was applied to remove 200 mg/L Pb^2+^ from water by immobilizing this strain on PES. The new biosorbent achieved a high removal rate of Pb^2+^, reaching 98%, compared to neat PES, confirming that the biosorbent is responsible for the adsorption process. Bacterial biomass possessed the PES membrane the adsorptive and hydrophilic properties. These novel biosorbents are separated easily from aqueous solutions and can be reused. Therefore, it could be stated that *P. dendritiformis* 17OS isolate seemed to be eco-friendly for removing heavy metals.

## Supplementary Information

Supplementary Information.

## Data Availability

The dataset used and analyzed during the current study is presented in the manuscript. The sequencing data generated and analyzed during the recent research are available in the NCBI Sequence Read Archive database https://www.ncbi.nlm.nih.gov/nuccore/On705726.1, Accession Number: ON705726.
