# Supplementary material for: An immobilized biosorbent from Paenibacillus dendritiformis dead cells and polyethersulfone for the sustainable bioremediation of lead from wastewater
Source: Sci Rep. 2023 Jan 17;13:891. doi: 10.1038/s41598-023-27796-w (PMC9845294; doi:10.1038/s41598-023-27796-w)
Supplement: Supplementary file 1 — Supplementary Information. [file 41598_2023_27796_MOESM1_ESM.docx]

**An immobilized biosorbent from *Paenibacillus dendritiformis* dead cells and polyethersulfone for the sustainable bioremediation of lead from wastewater**

[Ghada E. Dawwam](https://ejbo.journals.ekb.eg/?_action=article&au=358281&_au=Ghada+E.++Dawwam)^1^*, Nehad M Abdelfattah ^1^, Mohamed O. Abdel-Monem^1^, Hossam S. Jahin ^2^, Amal M. Omer^3^, Khadiga A. Abou-Taleb^4^ and Eman S. Mansor^5^

^1^ Botany and Microbiology Department, Faculty of Science, Benha University, Benha, 13518, Egypt.

^2^ Central Laboratory for Environmental Quality Monitoring, National Water Research Center, Elkanatir 13621, Egypt.

^3^ Desert Research Center, Department of Soil Fertility and Microbiology, El-Matareya 11753, Cairo, Egypt.

^4^ Department of Agricultural Microbiology, Faculty of Agriculture, Ain Shams University, P.O. Box 68, Hadayek Shubra 11241, Cairo, Egypt.

^5^ Water pollution Research Department, Environment Research and Climate Changes Institute, National Research Centre, Dokki, Cairo, Egypt.

*Corresponding author. [ghada.ibrahem@fsc.bu.edu.eg](mailto:ghada.ibrahem@fsc.bu.edu.eg). https://orcid.org/0000-0002-2911-658X.

**Materials and Methods**

**2.1. Metal solution preparation**

Heavy metal stock solutions were prepared according to Liu et al. ^22^ using a weighted quantity of heavy metal salts (HIMEDIA Co., Germany) containing 1 g nitrate salts of Pb(NO_3_)_2_, Cd(NO_3_)_2_, Cr(NO_3_)_3_, Zn(NO_3_)_2_, or Cu(NO_3_)_2_. These amounts were individually dissolved in a specific volume (100 ml) of deionized water to get a final concentration of 10,000 mg/L, which was used to produce different metals. Metal solutions were sterilized by filtration using sterile filters (0.2 µm pore size).

**2.2. Preparation of the standard inoculum**

The standard inoculum was prepared by inoculating the bacterial culture into 50 ml nutrient broth and incubating it on a rotary shaker at 150 rpm at 30 ± 2°C for 24 h. For shake flask tests, the contents of these flasks were used as the standard inoculum, with 1 mL containing 3.7 × 10^7^ colony-forming units/mL.

**Results**


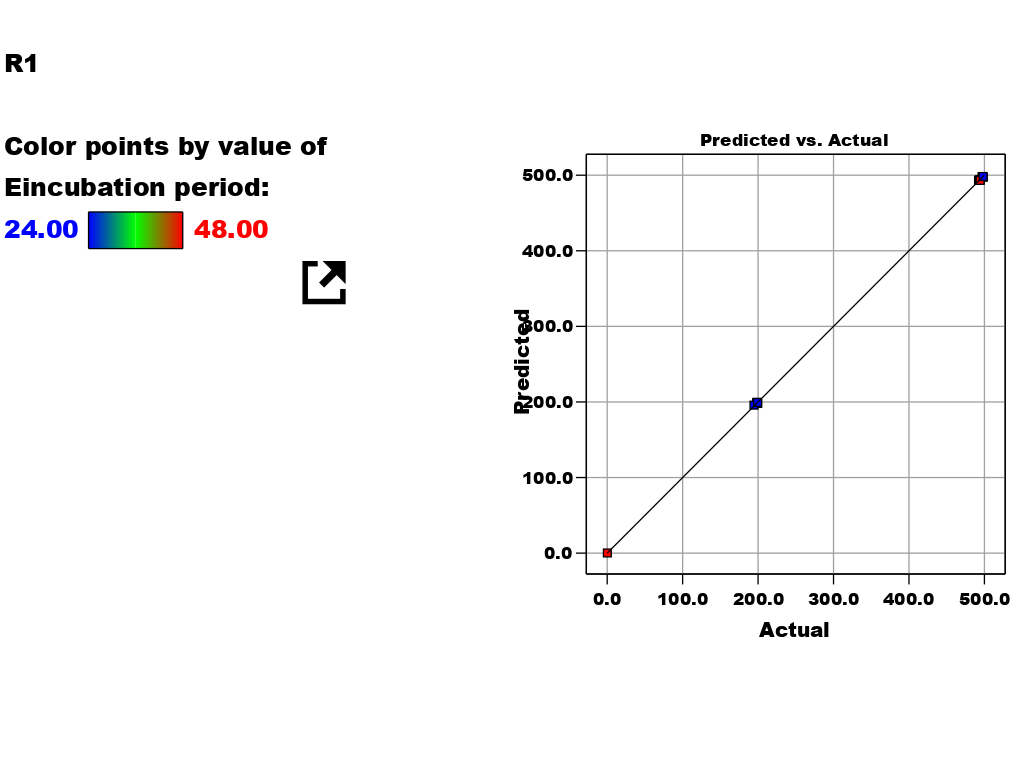

**Fig. S1** The actual and predicted values of the minimum run resolution IV experimental design for Pb^2+^ metal removal by 17OS isolate

**Fig. S2** The model graph for Pb^2+^ metal removal by 17OS strain at a one-factor level

**Fig. S3** The model graph for Pb^2+^ metal removal by 17OS strain at the interaction of the two-factor level


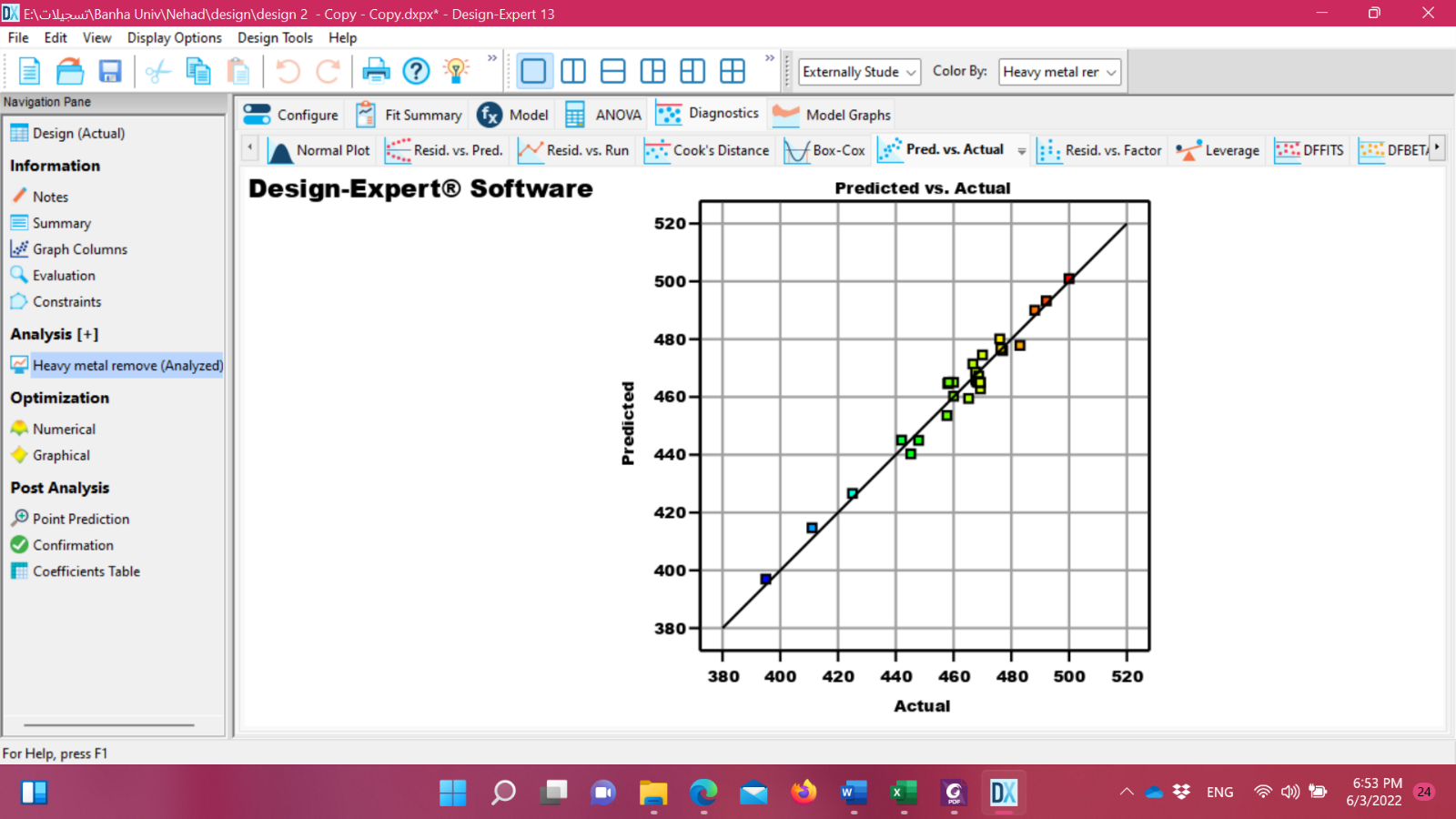


**Fig. S4** The actual and predicted values of Box-Behnken experimental design for Pb^2+^ metal removal by 17OS strain
